# Minimally Invasive Surgery in Gynecologic Oncology

**DOI:** 10.1155/2013/312982

**Published:** 2013-08-12

**Authors:** Kristina M. Mori, Nikki L. Neubauer

**Affiliations:** Division of Gynecologic Oncology, Department of Obstetrics and Gynecology, Northwestern University Feinberg School of Medicine, 250 E. Superior Street, Suite 05-2168, Chicago, IL 60611, USA

## Abstract

Minimally invasive surgery has been utilized in the field of obstetrics and gynecology as far back as the 1940s when culdoscopy was first introduced as a visualization tool. Gynecologists then began to employ minimally invasive surgery for adhesiolysis and obtaining biopsies but then expanded its use to include procedures such as tubal sterilization (Clyman (1963), L. E. Smale and M. L. Smale (1973), Thompson and Wheeless (1971), Peterson and Behrman (1971)). With advances in instrumentation, the first laparoscopic hysterectomy was successfully performed in 1989 by Reich et al. At the same time, minimally invasive surgery in gynecologic oncology was being developed alongside its benign counterpart. In the 1975s, Rosenoff et al. reported using peritoneoscopy for pretreatment evaluation in ovarian cancer, and Spinelli et al. reported on using laparoscopy for the staging of ovarian cancer. In 1993, Nichols used operative laparoscopy to perform pelvic lymphadenectomy in cervical cancer patients. The initial goals of minimally invasive surgery, not dissimilar to those of modern medicine, were to decrease the morbidity and mortality associated with surgery and therefore improve patient outcomes and patient satisfaction. This review will summarize the history and use of minimally invasive surgery in gynecologic oncology and also highlight new minimally invasive surgical approaches currently in development.

## 1. Laparoscopy in Cervical Cancer

### 1.1. Radical Hysterectomy

Laparoscopic surgery has played a role in the treatment of cervical cancer since the late 1980s. Nichols reported on laparoscopic lymphadenectomy for cervical cancer in 1993, over 30 years ago [1]. The laparoscopic radical hysterectomy with pelvic and para-aortic lymph node dissection was then first reported by Nezhat et al. a few years later [2]. When compared to the traditional radical hysterectomy performed via laparotomy, the laparoscopic approach allows for less blood loss and a shorter hospital stay at the cost of slightly increased procedure times. A retrospective study from Memorial Sloan Kettering compared 195 laparotomy patients to 17 laparoscopy patients undergoing radical hysterectomy. In this study, there was no significant difference between mean pelvic lymph node count (30.7 versus 25.5), transfusion rate (21 versus 5.3%), or negative surgical margins (5.1 versus 0%). The mean operating room times (296 versus 371 minutes, *P* < 0.01), mean EBL (693 versus 391 mL, *P* < 0.01), and mean length of hospital stay (9.7 versus 4.5 days, *P* < 0.01) were significantly different with a lower EBL and shorter hospital stay in the laparoscopic group, but a longer mean operating time in the laparoscopic group [3]. Another retrospective study, from MD Anderson, compared 54 laparotomy and 35 laparoscopic radical hysterectomies for cervical cancer. There was a significant difference in mean blood loss between the two groups (548 versus 319 mL), but no significant difference in transfusion rates (15 versus 11%). Again, the operative times were significantly longer in the laparoscopic group (344 versus 307 minutes), and the median length of stay was shorter in the laparoscopic group (5 versus 2 days, *P* < 0.001). The incidence of postoperative infectious morbidity including fever, wound cellulitis, urinary tract infection, pneumonia, intra-abdominal abscess, and necrotizing fasciitis was significantly greater in the patients undergoing laparotomy (53 versus 18% *P* < 0.001). The final pathology of the two groups showed no difference in the amount of resected parametrium, negative surgical margins, or lymph node metastasis. In this study, the total number of pelvic lymph nodes was significantly higher with the abdominal approach (18.7 versus 13.5 nodes, *P* < 0.001) [4]. More recently, Wright et al. published a study comparing 1610 abdominal approaches to 217 laparoscopic approaches. They found significantly higher rates of any complication (15.8 versus 9.2%, *P*  0.04), intestinal injuries (0.4 versus 1.8%, *P*  0.02), medical complications (8.8 versus 3.2, *P*  0.01), and blood transfusions (15 versus 5.1, *P* < 0.0001) in the abdominal group. A hospital length of stay longer than 3 days (44.3 versus 11.1, *P* < 0.0001) was also greater in the abdominal group. There was no difference in intraoperative complications (bladder, ureteral, intestinal, vascular, or other injuries) or surgical site complications (wound complications, abscess, hemorrhage, or bowel obstruction) [5].

Li et al. reported on a retrospective review of 35 open radical hysterectomies and 90 laparoscopy radical hysterectomies and also looked at recurrence rates. The follow-up from this study (a median of 26 months) showed no differences in the rate of recurrence (12 versus 13.75%, *P* > 0.05) or mortality between the two groups (8 versus 10%, *P* > 0.05) [6]. Nam et al. reviewed 263 cases of laparoscopic radical hysterectomy that were matched 1 : 1 with cases performed via laparotomy and found significant differences between the two groups in terms of EBL and hospital stay. As in Li's study, there was no significant difference in survival between the groups in regard to the 5-year recurrence-free survival rate (94.4 versus 92.8%, *P* = 0.499), 5-year overall survival rates (96.4 versus 95.2%, *P* = 0.451), and 5-year disease-specific survival rates (96.4 versus of 95.2% *P* = 0.387). There was no significant difference observed when the patients were divided into those with ≤2 cm tumors and those with >2 cm tumors, or when analyzed by age ≤46 years versus >46 years, tumor histology, and risk for recurrence [7]. From these studies we can conclude that a laparoscopic approach to radical hysterectomy for the treatment of cervical cancer is feasible and safe with less postoperative morbidity.

### 1.2. Trachelectomy

Radical vaginal trachelectomy for cervical cancer was developed and described by Daniel Dargent in 1987 as a fertility sparing surgery for women with cervical cancer [8]. This procedure was first described as a radical vaginal surgery, and surgeons have since then developed both abdominal and minimally invasive approaches to this procedure. Laparoscopy has become another modality by which to perform this procedure. Given how infrequently trachelectomies are performed, information about this procedure comes from case reports and case series. In 2003, two separate groups described this procedure being done in part laparoscopically [9, 10]. The first reported case of a total laparoscopic trachelectomy was in 2005 by Cibula et al., with no reported intraoperative or postoperative complications [11]. Park et al. reported on 4 cases of laparoscopic trachelectomies in 2009. One patient was stage IA2 and 3 were IB1, and all were squamous cell in histology. The mean surgical time was 250 min (range of 238–263 min), and the mean estimated blood was 185 mL (range of 60–280). There were no immediate postoperative complications, and after a mean followup of 34 months, there was one recurrence. All but one case had resumed menstruation, but there were no reported pregnancies [12]. 

In 2010, Kim et al. reported on 27 successful cases of laparoscopically assisted vaginal trachelectomy. Seventy-four percent of the tumors had a squamous histology while 22.2% were adenocarcinomas. All patients had negative resection margins, and the mean operating time was 290 min (range of 120–520). The mean estimated blood loss was 332 mL, and 6 patients (22%) did receive a transfusion. There were no intraoperative or postoperative complications and after a median follow-up time of the 31 months (range of 1–58), 1 patient had experienced a recurrence. Regular menstruation did resume in 24 patients; however, 8 patients reported decreased menstrual flow and 3 complained of new severe dysmenorrhea. Among the 6 patients attempting to conceive, 3 succeeded [13]. Martin and Torrent reported on 9 cases, similar to the Kim study, where the vaginal cuff incision and cervical reconstruction were performed vaginally. Six patients had squamous cell carcinoma, and 3 had adenocarcinoma. Two were stage IA1 and 7 were IB1. The mean operative time was 270 minutes, and all patients had negative surgical margins. The mean hospital stay was 5.2 days and the mean time for restoration of normal urinary function was 2 weeks. After a mean followup of 28 months (range 6–32), 4 patients had attempted pregnancy with 2 successes and one live full-term birth. There was 1 recurrence of adenocarcinoma 14 months posttrachelectomy that was treated with 3 cycles of cisplatin and paclitaxel and subsequent hysterectomy and radiation for eventual no evidence of disease status [14]. When comparing these laparoscopic cases to trachelectomies performed via a vaginal approach, it appears that there is no difference in recurrence or pregnancy rates. From the previous data we can conclude that a laparoscopic approach to trachelectomies for cervical cancer is a feasible option.

## 2. Laparoscopy in Endometrial Cancer

### 2.1. Hysterectomy and Staging

Historically, the surgical treatment of endometrial cancer has been performed via laparotomy. Laparoscopic technology has granted surgeons a method of treatment and staging in patients, who are likely to benefit the most given their tendency to have higher body mass indices and other associated comorbidities. The Gynecologic Oncology Group LAP2 Study randomized 2616 patients, in an approximately 2 : 1 fashion, to a laparoscopic versus open approach for the treatment and staging of endometrial cancer. The primary endpoint of this study was to compare recurrence free survival rates with secondary endpoints being the comparison of perioperative complications, conversion rates, and length of hospital stay. Twenty-five percent of the laparoscopy group were converted to laparotomy. The most common reason for conversion was poor visualization, but age >63, increasing BMI, and presence of metastatic disease all increased a patient's risk for conversion. The median operative time for the laparotomy group was 130 minutes versus 204 minutes for the laparoscopy arm (*P* < 0.001). The intraoperative complications (8 versus 10%), readmission rates (7 versus 6%), reoperation rates (2 versus 3%), and 30-day perioperative deaths (8 versus 10) were not significantly different between laparotomy and laparoscopy groups. Postoperative complications, including intestinal ileus, cardiac arrhythmia, antibiotic use, and hospital stay >2 days were significantly less likely in the patients undergoing a laparoscopic approach, occurring in 21% of the laparotomy group and 14% of the laparoscopy group (*P* < 0.001). With regard to staging, 97% of the laparotomy group had documented para-aortic lymph nodes in the final specimen, which was significantly different from 94% of the laparoscopy group (*P* = 0.002) [15]. After a median of 59.3 months of followup for both groups, there were a total of 309 recurrences (210 laparoscopy, 99 laparotomy) and 350 deaths (229 laparoscopy, 121 laparotomy). The 3-year estimated cumulative incidence of recurrence for laparotomy patients was 10.24%, compared with 11.39% for laparoscopy patients, with a hazard ratio of 1.14 (CI −1.278–3.996). There was no difference in the estimated 5-year overall survival (89.9% in each group), postoperative adjuvant therapy, and site of recurrence [16]. From this important study, we can conclude that a minimally invasive approach to the treatment of endometrial cancer is as good as an open approach with many benefits including fewer postoperative complications, a shorter hospital stay, and less blood loss.

The Cochrane Collaboration published a review in 2012 that included 8 studies, of which at least 70% of patients had early stage endometrial cancer; the 2009 Walker study previously cited was included. When comparing laparoscopy to laparotomy, the review concluded that there were no differences in overall survival (HR 1.14, CI 0.62–2.10), recurrence-free survival (HR 1.13, CI 0.90–1.42), or perioperative death (HR 0.76, CI 0.3–1.79) between the two groups. The estimated blood loss was lower in the laparoscopy group (mean difference of −106.82 mL, 95% CI: −141.59, −72.06), though the need for blood transfusion was not significantly different (95% CI: 0.21, 1.49). There was also no significant difference of bladder injury (RR = 0.49, 95% CI: 0.13, 1.86), bowel injury (RR = 1.49, 95% CI: 0.39, 5.72) or vascular injury (RR = 0.43, 95% CI: 0.08 to 2.32) between patients undergoing laparoscopy and laparotomy. The risk of severe postoperative complications was significantly lower with laparoscopy with a relative risk of 0.58 (95% CI: 0.37 to 0.91) [17]. Given the available data for the use of laparoscopy in endometrial cancer, laparoscopy seems to have significant perioperative and postoperative benefits in these patients without sacrificing the desired oncologic outcomes.

## 3. Laparoscopy in Ovarian Cancer

Laparoscopy has also been reported on for staging in early ovarian cancer. Chi et al. reported a case-control study of 20 patients undergoing laparoscopy and 30 patients undergoing laparotomy. Baseline characteristics of age, BMI, primary disease site, histology, and tumor grade did not differ between the groups; however, 65% of the patients undergoing laparoscopy had a cancer diagnosis prior to surgery compared to only 23% of the laparotomy patients (*P*  0.003). There was no significant difference between laparoscopy and laparotomy in terms of the number of lymph nodes removed, the size of the omental specimen, the site of metastases, or complications. The mean operating times (321 versus 276 minutes, *P*  0.04), mean estimated blood loss (235 versus 367 mL, *P*  0.003), and length of hospital stay were significantly different (3.1 versus 5.8 days, *P* < 0.001) favoring the laparoscopic group. Three postoperative complications (2 wound infections and 1 ileus) were reported, all of them in the laparotomy group [18]. Park et al. reported on a similar study that included 17 laparoscopic patients and 19 laparotomy patients. All patients in the laparoscopy group had previously undergone abdominal surgeries compared to 57.9% in the laparotomy group (*P*  0.013). There was no difference in the mean number of lymph nodes removed or time to adjuvant chemotherapy. The laparoscopy group differed significantly from the laparotomy group in regard to mean estimated blood loss (231 versus 505 mL, *P*  0.001), mean number of days to the return of bowel movements (2 versus 3.8, *P* < 0.001), and mean postoperative stay (9.4 versus 14.1 days, *P*  0.002). There were 2 recurrences after a median followup of 17 months in the laparoscopy group but there was no difference in disease-free or overall survival between the groups [19]. Nezhat et al. looked at 32 women with gross extraovarian disease who all had their procedures started laparoscopically, but then, at surgeon discretion, they were placed in 1 of 3 groups: primary cytoreduction and interval debulking via laparoscopy (17 patients), primary cytoreduction and debulking via laparotomy (11 patients), or biopsies only (4 patients). The biopsy group included 2 primary gastrointestinal cancers, 1 benign struma ovarii, and 1 primary peritoneal adenocarcinoma that declined debulking. All patients in the first group were stage IIIA or greater and 88.2% had optimal cytoreduction. The patients of the second group were stage IIIB or greater. Groups 1 and 2 did not differ with regard to mean operative time, intraoperative, or postoperative complications. There was a significant difference between Group 1 and Group 2 in regard to mean estimated blood loss (247 versus 609, *P*  0.008) and mean days of hospital stay (6.1 versus 8.2, *P*  0.03). The median time to recurrence for Group 1 was 31.7 months and 21.5 months for Group 2; however, this did not meet statistical significance (*P* = 0.3) [20]. It appears that a laparoscopic approach is also feasible in the treatment of ovarian cancer, particularly in early stage disease, but more data is needed regarding long-term oncologic outcomes of these patients.

## 4. Robotics in Cervical Cancer

### 4.1. Radical Hysterectomy

Yet another minimally invasive approach that has gained popularity over the last several years is the use of robotic surgery in the treatment of gynecologic cancers. With the introduction of the robotic platform, there is now another modality by which to perform a radical hysterectomy. Lowe et al. reported in 2009 on 42 patients who underwent a robotic-assisted radical hysterectomy. Stage of disease ranged from IA1 with lymphovascular space invasion to IB2. The median operative time was 215 minutes, median estimated blood loss was 50 cc, median lymph node count was 25, and the median hospital stay was 1 day. All patients had negative parametrial and vaginal margins, but 12% had evidence of positive lymph nodes. There were 2 intraoperative complications (4.8%) that included 1 conversion to laparotomy to repair a cystotomy and 1 ureteral injury. Postoperatively, DVT occurred in 2.4% of the subjects, pyelonephritis in 2.4%, and infection in 4.8%. There were no readmissions or reoperations [21]. Cantrell et al. evaluated 63 robotic cases and compared outcomes to open radical hysterectomies and found some significant differences between the 2 groups perioperatively. When the robotic cases were compared to the laparotomy cases, there was a lower mean estimated blood loss (50 versus 400 mL, *P* < 0.0001), a higher median number of lymph nodes (29 versus 24, *P*  0.04), shortened operative time (213 versus 240 min, *P*  0.0015), and shorter hospital stay in the robotic population (1 versus 4 days, *P* < 0.0001). After a median followup of 12.2 months in the robotic group and 28 months in the laparotomy group, there was no apparent difference in progression-free or overall survival [22]. Geetha and Nair have reviewed 12 studies of robotic radical hysterectomies (including 327 patients) and 14 studies of open radical hysterectomies (1552 patients). In all studies, there was actually no statistical difference in the mean operative time. Concurrent with the above study by Cantrell et al., the mean blood loss was significantly lower in the robotic cases, the mean hospital stay was significantly lower in the robotic group, and the percentage of patients with infectious perioperative morbidity was significantly higher in the laparotomy group. With regard to oncologic outcomes, the mean nodal metastases, positive margins, and recurrence rates were not different between the groups [23]. It appears that a robotic approach to radical hysterectomy for the treatment of cervical cancer is feasible and affords the same staging abilities as open surgery with less blood loss and a shorter hospital stay.

### 4.2. Radical Trachelectomy

Using robot assistance for a fertility-sparing surgery in early cervical cancer is reported in a number of case reports and case series [24–29]. Persson et al. recently published a retrospective cohort comparing 13 cases of robotic trachelectomy to 12 vaginal cases. The stage of disease was similar between the two groups with 4 women with stage IA1 cervical cancer in the vaginal group versus 4 in the robotic group, 2 in the vaginal group versus 5 in the robotic group with stage IA2, respectively, and 6 in the vaginal group versus 4 in the robotic group with stage IB1 cervical cancer, respectively. Two cases in the vaginal group and 1 in the robotic group were converted to radical hysterectomies due to close proximal margins or positive lymph nodes. The mean operative times for both groups were not significantly different (297 minutes in the robot group versus 254 minutes in the vaginal group; *P*  0.26). The robotic group did have significantly lower estimated blood loss (133 versus 289 mL; *P*  0.05) and shorter hospital stay (2.3 versus 3.6 days; *P*  0.02). There were no reported recurrences in either group. With regard to fertility, 5 women in the robotic group and 8 in the vaginal group actively attempted pregnancy postoperatively. Four women in the robotic group were successful, with 2 reported deliveries, and 7 women in the vaginal group were successful, with 10 births [30].

### 4.3. Exenteration

The morbidity of pelvic exenteration is reported to be as high as 50–60% with a 5–7% mortality rate [31]. Robotic-assisted surgical techniques may help to decrease these associated morbidities. Although the approach is novel, there are a handful of case reports in the literature that confirm safety of the procedure and possible decrease in estimated blood loss and hospital stay for select patients [32–34].

## 5. Robotics in Endometrial Cancer

Given the high conversion rate in GOG LAP2, it seems prudent to search for a minimally invasive approach to endometrial cancer that can be utilized in older, obese patients. Robotics may solve some of the technical difficulties associated with laparoscopy including improved physician ergonomics. Lowe et al. reported on 405 patients from multiple medical centers who underwent robotic-assisted hysterectomy and staging for endometrial cancer. The mean operative time was 170.5 minutes, estimated blood loss was 87.5 mL, length of hospital stay was 1.8 days, and lymph node count was 15.5. The conversion rate to laparotomy was 6.7% with reasons such as grossly involved adnexa or nodal disease or uterine size greater than anticipated being cited as major reasons for conversion. Intraoperative complications were rare at 3.5% and postoperative complications occurred in 14.6% of patients, the majority of which were fever, urinary tract infection, DVT, and wound seroma [35]. In 2012, Gaia et al. published a meta-analysis reviewing 589 robotic-assisted surgeries, 396 laparoscopic surgeries, and 606 open surgeries. When compared to laparoscopy, robotic-assisted surgeries had a lower estimated blood loss (91.6 versus 182 mL, *P* < 0.001). Otherwise, there was no statistical difference in hospital length of stay (1.35 versus 1.9 days), number of aortic lymph nodes (10.3 versus 7.8), number of pelvic lymph nodes (18.5 compared with 17.8), operative times (219 versus 209 minutes), wound complications (2% versus 2.8%), rate of conversion to laparotomy (4.9% versus 9.9%), or “other” complications (stroke, ileus, lymphedema, nerve palsy, acute renal failure, lymphocyst, and urinary retention) (2 versus 3.8%, OR 0.54, CI 0.16–1.81, *P*  0.23). When compared to laparotomy, robotic-assisted surgeries had a longer mean operative time (207 versus 130 minutes, *P* < 0.005), a lower mean estimated blood loss (101 versus 291 mL, *P* < 0.005), a shorter average hospital stay (1.2 versus 3.9 days, *P* < 0.001), a lower rate of wound complications (1.8 versus 13.7%, OR 0.13, CI 0.04–0.44. *P*  0.01), and a lower rate of “other” complications (3.8 versus 14.5%, OR 0.25, CI 0.10–0.60, *P*  0.01). There was no difference in the number of pelvic or aortic lymph nodes retrieved when Robotic-assisted surgeries were compared to laparotomy (18.0 versus 14.5 and 9.4 versus 5.7, resp.). Across all groups, there was no statistical difference in vascular, bowel, and bladder injuries, vaginal cuff dehiscence, thromboembolic events, or an unplanned return to the operating room for bleeding [36].

When evaluating the utility of robotics in obese endometrial cancer patients specifically, there appears to be significant advantages. Subramaniam et al. compared 73 obese women who underwent robotic surgery to 104 obese women who underwent laparotomy, though 8 patients in the robotic cohort underwent conversion to laparotomy (11%). The rates of lymphadenectomy between the two groups were similar (65.8% of the robotic group versus 56.7% of the laparotomy group, *P*  0.227), as was the mean number of lymph nodes removed (8.01 versus 7.24, *P*  0.505). The mean operative time from skin opening to closure was significantly longer in the robotic group (246.2 versus 138.2 min, *P* < 0.001), as was the mean time in the operating room (303.2 versus 191.4 min, *P* < 0.001). The mean estimated blood loss (95.9 versus 408.9 cc, *P* < 0.001), percentage of patients receiving a blood transfusion (1.4% versus 13.5%, *P*  0.005), mean length of hospital stay (2.73 versus 5.07 days, *P* < 0.001), wound complications (4.1% versus 20.2%, *P*  0.002), and non-wound complications (including cardiac, pulmonary, and gastrointestinal causes) (9.6% versus 29.8%, *P*  0.001) were significantly lower in the robotic group [37]. Gehrig et al. also specifically looked at higher BMI patients and compared 49 obese and morbidly obese women who underwent robotic surgery to 32 obese and morbidly obese women who underwent traditional laparoscopy. When compared to the laparoscopy group, the robotic group had a significantly lower mean operative time (189 versus 215 minutes, *P*  0.0004), mean estimated blood loss (50 versus 150 mL, *P* < 0.0001), and mean hospital stay (1.02 versus 1.27 days, *P*  0.01). The mean paraaortic and total lymph node counts were greater in the robotic group (10.3 versus 7.03, *P*  0.01; 31.4 versus 24, *P*  0.004); however, this difference was not seen in the morbidly obese patients alone. There were 2 conversions to laparotomy in the laparoscopy cohort and none in the robotic cohort, and there was no difference in the completion of comprehensive surgical staging between the two groups. There was also no difference in the rates of operative or postoperative complications [38].

Backes et al. recently published on the short- and long-term morbidities of robotic surgery in 503 endometrial cancer patients after a median followup of 25 months. Ninety-three percent of these patients underwent a pelvic lymph node dissection during their primary surgery. The conversion rate to laparotomy was 6.4%, with dense adhesions, poor visualization, and bleeding listed as the top reasons for conversion. Intraoperatively, there were two enterotomies, one ureteric injury, and five vessel injuries. Postoperatively, 11 patients developed an ileus (16% of the group that was converted to laparotomy and 1.3% of the robotic patients), and 21 patients developed a wound complication (18.8% of the converted group and 3.2% of the robotic group). The rate of postoperative venous thromboembolism was 2.2%, and the rate of postoperative fever was 2.4%. One patient developed a femoral neuropathy, 21 developed genitofemoral neuropathies, 1 developed a lateral femoral cutaneous neuropathy, 1 developed a peroneal nerve injury, and 2 developed mild obturator nerve injuries. The rate of cuff dehiscence was 2.4%. Postoperative lymphedema occurred in 12.7% of patients, with only 1.8% reporting severe symptoms. Forty-five (8.9%) patients were readmitted with surgery related complications, with the primary reasons being vaginal cuff complications followed by ileus [39]. From this study we can counsel patients about the morbidity rates associated with robotic surgery for endometrial cancer and conclude that the overall intraoperative and postoperative complication rates following robotic surgery are low.

Brudie et al. reported on the recurrence-free survival and overall survival of 372 patients who underwent robotic surgery after a median followup of 31 months. Adjuvant therapies were not standardized but directed by physician preference. The risk of recurrence for all patients was 8.3%, with 4.6% of patients dying of their disease. The estimated 3-year recurrence-free survival for the entire group was 89.3% with an estimated 5-year overall survival of 89.1% and 92.5% and 93.4% for the endometrioid subset. These results appear very similar to those of the LAP2 study, reinforcing the idea of that disease outcomes are not altered when robotic assistance is used for endometrial cancer surgery [40]. The use of robotics in the treatment of endometrial cancer seems promising with similar outcomes as laparoscopy and may bridge the gap between those patients who would otherwise not be treated with a minimally invasive approach due to either patient comorbidities or surgeon skill level.

## 6. Robotics in Ovarian Cancer

### 6.1. Debulking

Holloway et al. describes the utility of robotic assistance in a patient with recurrent platinum-sensitive ovarian cancer who had a metastasis to her liver that was persistent after chemotherapy. They succeeded in a complete resection with negative margins in a total operating room time of 137 minutes and 100 mL estimated blood loss [41]. Magrina et al. compared 35 patients undergoing primary surgical treatment for ovarian cancer who underwent a robotic-assisted surgery to matched cohorts of patients who underwent laparoscopic and open procedures for the treatment of ovarian cancer. All groups were separated into 3 subgroups, depending on the extent of their surgery. Type I patients underwent a hysterectomy, an adnexectomy, an infracolic or infragastric omentectomy, a pelvic and aortic lymphadenectomy, an appendectomy, and the removal of metastatic peritoneal disease if it was present. Type II patients underwent a Type I debulking and 1 additional major procedure. Type III patients underwent a Type I debulking and 2 or more major procedures. Major procedures were described as any type of intestinal resection (modified posterior pelvic exenteration with low colorectal anastomosis, sigmoid resection with high anastomosis, transverse colon resection, ileocecal resection, and/or small bowel resection), full thickness diaphragm resection, resection of liver disease, and splenectomy. Of note, there were now laparoscopic Type III surgeries reported. Complete or incomplete debulking was based on whether there was visible residual tumor of any size at the conclusion of the case. Presence of FIGO stage III-IV disease was 60%, 75%, and 87% for robotics, laparoscopy, and laparotomy, respectively. The mean operating time was significantly longer in the robotic group when compared to the laparoscopic and laparotomy groups (315 versus 254 versus 261 minutes, *P* < 0.05), except in the Type I surgeries (282 versus 249 versus 230 minutes, *P*  0.10). As expected from previous surgical reports, the mean estimated blood loss was significantly lower in the robotic and laparoscopic groups in comparison to the open group (164 versus 267 versus 1307 mL, *P* < 0.05). The overall mean hospital stay was much lower in the robotic and laparoscopic groups compared to laparotomy (4 versus 3 versus 9 days, *P* < 0.05). However, the length of stay for the Type III surgical patients did not differ between the robotic and open approaches (mean 11 versus 10 days). There was no statistically significant difference in intraoperative complications among the 3 groups in all 3 surgery types. Postoperative complications (within 42 days) were similar among all groups with a Type I surgery, lower for robotics and laparoscopy patients with a Type II surgery (25 versus 0 versus 54%, *P*  0.01), then similar between the robotic and laparotomy groups with a Type III surgery (100 versus 56%). The rate of complete debulking was greater in the robotic and laparoscopic arms than in the open arm (84 versus 93 versus 56%, *P* < 0.001). However, this is likely attributable to surgeon surgical preference, choosing an open method for those with more disseminated disease. Twenty-four, 29.6, and 24.3% of patients in the robotic, laparoscopic, and laparotomy groups received neoadjuvant chemotherapy followed by 3 courses of postoperative chemotherapy on GOG 172. The remaining patients all received adjuvant chemotherapy following primary debulking, except for 4 laparotomy patients who did not, due to 1 postoperative death, and 3 with prolonged postoperative complications and older age. The 3-year overall survival between groups was no different, even when comparing stage and surgery type. The 3-year progression free survival, however, was lower overall in the laparotomy group when compared to the robotic and laparoscopic groups (40.2 versus 74.2 versus 62.6%, *P*  0.003). The authors hypothesized that this was due to surgeon selection for an open approach in patients with more disseminated disease, as well as a higher percentage of patients in the laparoscopy and robotic groups having undergone neoadjuvant or adjuvant IP chemotherapy (40.7% versus 48% versus 36.5%). When comparing cancer stage, this difference remained statistically significant among the stage III-IV patients (30 versus 55.6 versus 48.5%, *P*  0.03). A valuable finding of this study is that it appears that disease state and complete debulking are more important in determining prognosis than the surgical approach [42]. At our institution we are currently performing interval debulking surgeries with a robotic approach and have noted good outcomes with ability to remove all gross disease while decreasing blood loss and hospital stay associated with the procedure. It appears that patients with early stage ovarian cancer undergoing complete staging procedures and those undergoing neoadjuvant surgeries may benefit most from a robotic approach; however, the robotic approach may also be feasible for certain patients with stage III and greater disease undergoing a primary debulking procedure. 

### 6.2. Adnexal Masses

Magrina et al. has looked at 85 patients who underwent robotic-assisted surgery for adnexal surgery and compared them to 91 patients who underwent traditional laparoscopy. In the robotic group, the indication for surgery was an adnexal mass in 90% and prophylactic oophorectomy in 10% of patients. In the laparoscopy group, the indications were similar with 97% undergoing surgery for an adnexal mass and 3% undergoing prophylactic surgery. Demographically, the robotic group had a statistically higher number of obese patients (35 versus 18%, *P*  0.02), higher number of patients with an American Society of Anesthesiologists (ASA) physical status classification of 2 or 3 (45 versus 27%, *P*  0.04), and a higher number of patients who underwent a unilateral salpingo-oophorectomy (26 versus 3%, *P*  0.003). The mean operating time was significantly longer in the robotic group by 12 minutes (83 versus 71 minutes, *P*  0.01). This difference in operating times was not seen among obese patients (BMI of 30 or more) (80 versus 71 minutes, *P*  0.43). The mean estimated blood loss between the two groups was similar (41 versus 39 mL, *P*  0.65), except in the obese patients, where the blood loss was higher in the laparoscopy group (60 versus 39 mL, *P*  0.02). There was no significant difference in intraoperative or postoperative complications between the 2 groups, and no cases were converted to laparotomy [43]. It appears from this study that obese patients may benefit the most from a robotic surgical approach to an adnexal mass, but there does not appear to be a significant difference in terms of complications and outcomes between a robotic and laparoscopic approach.

## 7. Laparoendoscopic Single-Site Surgery (LESS)

Laparoendoscopic single-site surgery was first described back in 1973 by Wheeless and Thompson for their tubal sterilization technique [44]. It was not until 1991 that Pelosi and Pelosi reported on its use to complete a total laparoscopic hysterectomy and bilateral salpingo-oophorectomy [45]. Following these reports, the interest in such an approach waned, likely due to the difficulties of such procedures given the available technology at the time. Likely secondary to the advent of more sophisticated technologies, the LESS approach seems to have just recently gained momentum among gynecologic surgeons. These procedures are characterized by a single incision, very often through the umbilicus, through which either multiple ports are placed or a single-port which can accommodate multiple ports and instruments.

With its newly gained popularity, the descriptions for this surgical approach have varied from OPUS (one port umbilical surgery) to SILS (single-incision laparoscopic surgery) to SPICES (single-port incisionless conventional equipment-utilizing surgery). In order to clarify surgeon communication and the research language, the Laparoendoscopic Single-Site Surgery Consortium for Assessment and Research (LESSCAR) published a consensus statement in 2010 establishing the term laparoendoscopic single-site surgery (LESS) as the standard term to describe such surgery.

Possible benefits of LESS are that with fewer surgical sites come superior cosmesis, decreased morbidity related to intra- and postoperative complications, possible decreased pain postoperatively, and faster postoperative recovery. A definite disadvantage has been lack of instrument triangulation and crowding. A solution to these issues has been developments of new articulating and/or flexible instruments and cameras. Another potential obstacle is increased cost with the need to use newer devices without a clear savings in the intra- or postoperative period for patients or society as compared to laparoscopy alone.

## 8. LESS in Gynecologic Oncology

Fader and Escobar first reported on the use of LESS in gynecologic oncology in 2009. This series included 13 patients, of whom 9 were performed on via LESS and 4 were with robotic-assisted LESS. One patient had staging for endometrial cancer, 1 had staging for granulosa cell ovarian cancer, 1 had a retroperitoneal pelvic lymph node dissection and peritoneal biopsies for a suspected right pelvic sidewall recurrence of papillary serous ovarian carcinoma, 2 had a risk-reducing extrafascial hysterectomy and bilateral salpingo-oophorectomy, 5 had a risk-reducing BSO alone, 1 had an ovarian cystectomy for a mature cystic teratoma, and 2 had bilateral salpingo-oophorectomies for complex adnexal masses. There were no conversions to conventional multiport laparoscopy or open surgery, no postoperative complications, and no early port-site hernias noted. The median overall operating time was 65 min (range 35–178), but the median operating time for hysterectomy with or without a lymphadenectomy was significantly longer at 168 min (range 145–178 minutes). The mean hospital stay was 0.7 days. Eighty-five percent of patients reported pain scores of 0-1 in the immediate postoperative period and at their follow-up visits, and 62% (including 2 of the 3 patients who underwent hysterectomies) reported not using narcotics at all as an outpatient. Surgeons attributed lack of instrument crowding in their cases to a laparoscope with a flexible tip and articulating instruments. Participating surgeons also determined that the surgical range of motion was increased in robotic cases when the Gelport was used as the access platform [46].

Fader et al. followed-up the above noted study with a report on a larger and multi-institutional retrospective series of patients. They reported on 74 attempted LESS gynecologic procedures, of which 96% were performed successfully through a single umbilical incision. Two endometrial cancer patients were converted to open and conventional laparoscopies due to adhesions, and 1 patient with ovarian cancer was converted to laparotomy due to metastatic pelvic implants. Indications for surgery were benign pelvic mass in 39 patients, endometrial hyperplasia in 9, endometrial cancer in 15, ovarian cancer in 6, and nongynecologic malignancies in 5 (breast, pancreatic, and lymphoma). All necessary staging was completed as indicated among the group. The median number of pelvic and para-aortic lymph nodes removed for endometrial and ovarian cases was 9 (range 7–21) and 3 (range 2–6). The mean hospital stay for patients with benign pathology was 0 days and 1 day for patients with malignancy. There were no intraoperative complications, and 3 perioperative complications were noted (1 pulmonary embolus after LESS for benign disease, 1 incisional cellulitis with malignant disease, and 1 cuff dehiscence with malignant disease). The median operative time for cancer staging was 132 minutes and 43 minutes for the nonstaging surgeries. In regard to analgesia use, patients with cancer required significantly more intravenous and oral narcotics to achieve adequate pain control as compared to the other patients (*P*  0.009). The authors hypothesized that this could be biased by the fact that cancer patients were more likely to spend at least 1 evening in the hospital. When questioned later, 42% of the benign patients and 30% of the cancer patients reported no outpatient narcotic use [47]. From these small studies it appears that LESS may be a useful surgical intervention in patients who desire a certain cosmetic outcome and may be better at reducing postoperative pain in patients with benign disease as opposed to a malignant etiology for their procedure. 

### 8.1. Endometrial Cancer

Three separate studies have evaluated the use of LESS in the treatment of endometrial cancer specifically. Fagotti et al. looked at 100 cases using LESS for total hysterectomy and bilateral salpingo-oophorectomy with and without lymph node dissection in stage I patients. The types of ports utilized in this study included the TriPort (*N* = 70), SILS port (*N* = 25), and Applied GelPoint Port (*N* = 5). Approximately half of the cuff closures were completed laparoscopically and half vaginally (48 versus 52 patients, resp.). The median estimated blood loss was 70 mL (range 10–500), median operative time was 129 minutes (range 45–321), and the median hospital stay was 1 day (range 1–4). The median estimated blood loss was significantly higher in the groups who had a lymph node dissection as compared to those who did not (100 versus 30 mL, *P*  0.0001). Also, not surprisingly, the median operative was also longer in the lymph node dissection groups when compared to the hysterectomy and BSO alone group (192 minutes with pelvic and aortic lymph nodes versus 142 minutes with pelvic lymph nodes versus 98 minutes without lymph nodes, *P*  0.0001). The median numbers of pelvic and para-aortic lymph nodes obtained were 16 (range 1–33) and 7 (range 2–28), respectively. There were no conversions to multiport laparoscopy or laparotomy for the hysterectomy and BSO patients. However, during lymphadenectomy, 1 patient was converted to laparotomy for an obturator nerve reapproximation after transection. There were 2 intraoperative complications (1 bowel and 1 vascular) that were managed via LESS and 1 vascular complication that needed conversion to conventional laparoscopy for repair. Postoperatively, there was 1 case of cellulitis at the wound site, 2 partial vaginal cuff dehiscences, and 1 ileus. Eighty-eight patients were evaluated on their satisfaction with the appearance of the surgical scar using a subjective score from 0–10 (0 being “bad” and 10 being “excellent”), and by 30 days the median value was 9 (range 8-9) [48].

Fanfani et al. reported on 20 prospectively collected patients with stage I endometrial cancer who underwent LESS surgery. This study differed from Fagotti's in that women with BMI > 35 were not included. Also, the 2 women who required lymphadenectomy were converted to traditional laparoscopy to complete their cases. The median EBL in this group was 20 mL (range 10–180) with a median operative time 105 min (range 85–155). There were no reported intraoperative or postoperative complications, and the median time to discharge was 1 day (range 1-2). By 30 days postoperatively, the median value of patient satisfaction with cosmetic outcome on a subjective score from 0–10 was 9 (range 8-9) [49]. From these two studies one can conclude that hysterectomy with bilateral salpingo-oophorectomy can be performed via LESS for the treatment of endometrial cancer; however, if other staging procedures are necessary, conversion to conventional laparoscopy or laparotomy may be required. 

Escobar et al. published a matched retrospective cohort study that compared 30 patients in each group of LESS, traditional laparoscopy, and robotic approaches for the treatment of endometrial cancer. There were no significant differences in operating times or estimated blood loss between the groups. When comparing the robotic group to LESS and traditional laparoscopy groups, the robotic group had a significantly higher median number of total pelvic lymph nodes (17 versus 16 versus 13 respectively, *P*  0.04). There was not a significant difference in the number of aortic lymph nodes removed, even though a significant portion of each group had them completed (33.3% robotic, 55% LESS, and 30% traditional laparoscopy). The LESS group had 1 conversion to traditional laparoscopy for extensive adhesions, and the traditional laparoscopy group had 1 conversion to laparotomy for a bowel and bladder injury [50]. Again, it appears that LESS can be useful for patients with endometrial cancer undergoing a hysterectomy and bilateral salpingo-oophorectomy but is a perhaps more challenging surgical approach for those patients that require lymph node sampling, particularly obese patients with a BMI > 35. 

### 8.2. Ovarian Cancer/Risk-Reducing Therapy

There are no studies evaluating the use of LESS in the treatment of ovarian cancer. Escobar et al. have reported on the use of LESS in 58 risk-reducing salpingo-oophorectomies (RRSO) among BRCA mutation carriers, women with breast cancer, and others at high risk for breast, ovarian, and/or endometrial cancer. Thirteen of the patients also underwent a total laparoscopic hysterectomy as part of their treatment. There were no conversions or intraoperative complications. Postoperatively, there were 2 partial vaginal cuff dehiscences and 1 periumbilical wound cellulitis. The mean operating time for those undergoing RRSO alone was 35 minutes (range 16–80). Seventy-five percent were discharged the day of surgery and 22% spent 1 night in the hospital. Two weeks postoperatively, only 69% required narcotics postoperatively, of which 62% (36 of 58) only required oral narcotics [51].

### 8.3. Lymphadenectomy/Staging

One series has been published by Escobar et al. describing the use of LESS surgery in staging gynecologic cancers after initial diagnostic surgery. In this series, 14 had endometrial cancer, 3 had a sex-cord stromal tumor/dysgerminoma, 1 had epithelial ovarian cancer, and 3 had locally advanced cervical cancer. Eleven of the endometrial cancer cases underwent a pelvic lymph node dissection, and the other 3 had pelvic and high para-aortic lymph nodes completed. Of this group, 1 had a small vascular injury requiring pressure and suturing, and 1 patient was converted to conventional laparoscopies due to dense adhesions. Again, no postoperative complications were noted. The authors did acknowledge the difficulty in performing the para-aortic lymphadenectomy in the morbidly obese patients in the group and utilized a lateral position with the left flank elevated to improve visualization and feasibility of completion. This is a common positioning method for transperitoneal laparoscopic left nephrectomy surgeries that may prove useful for the gynecologic oncologist performing a LESS para-aortic lymphadenectomy [52].

### 8.4. LESS and Robotics

With the known difficulties of the LESS approach and the significant rates of obesity, particularly in the United States, there has been an initiative to incorporate robotics in an attempt to overcome some of these difficulties. Escobar et al. first reported on the use of robotic-assisted LESS surgery in a case report of a 60-year-old BRCA-positive woman who underwent a risk-reducing total hysterectomy and bilateral salpingo-oophorectomy. The authors acknowledged the frustration of instrument crowding even with the use of robotic in this LESS setting [53]. Since the exploration of using robotics in combination with LESS, a single-site platform has been developed and tested in porcine and cadaver models with excellent results. Escobar et al. reported that the dedicated single-site platform allowed for “technically challenging procedures within acceptable operative times and without complications or insertion of additional trocars” [54, 55]. The development of technology to facilitate LESS robotic surgery may allow this approach to become more universal and especially useful in the obese patient with endometrial cancer who requires a full lymph node dissection.

## 9. Orifice-Assisted Small-Incision Surgery (OASIS)

Another novel approach to ease the frustrations of instrument crowding in LESS has been recently described by Einarsson et al. and is referred to as orifice-assisted small-incision surgery or OASIS. In this series, a port was placed through the posterior cul-de-sac and a flexible endoscope then used for optical access. Five patients had their surgery completed in Boston, and their indications were a symptomatic myomatous uterus and/or abnormal uterine bleeding. Their surgeries totalled 2 total laparoscopic hysterectomies, 2 laparoscopic supracervical hysterectomies, and 1 laparoscopic myomectomy. The median procedure time was 90 minutes (range 79 to 150), and the estimated blood loss ranged from 30 to 500 mL. No intra- or postoperative complications were reported. However, there were 2 procedures, in which laparoscopic suturing was performed, that required an additional 3- or 5 mm instrument in the left lower quadrant to enable triangulation. The other 9 patients have had their surgery in India. Their indications were either benign (*N* = 2) or oncologic (*N* = 5 cervical cancer stage IB1, *N* = 2 endometrial cancer stage IA), and procedures included 3 total laparoscopic hysterectomies and 6 laparoscopic radical hysterectomies with pelvic lymph node dissection. The overall median operative time was 110.10 minutes but 65 minutes for total laparoscopic hysterectomy and 130.0 minutes for laparoscopic radical hysterectomy. The mean estimated blood loss was 48.57 mL (range 10–100). All margins were negative in the cancer patients, and the lymph node count was 16 to 18. There was 1 complication of a ureteral leak during a radical hysterectomy that was treated with a stent [56].

### 9.1. Discussion

In conclusion, minimally invasive surgical techniques in gynecologic oncology have evolved greatly since the introduction of laparoscopy into the field. The advantages of laparoscopy over laparotomy, in the appropriately selected cancer patient, have proven benefits to the patient both intraoperatively and postoperatively, with similar outcomes. The fundamental aspects that are perhaps keeping this surgical approach from becoming more widespread are resources and surgeon skill and comfort level to complete extensive staging procedures. The advent of robotic-assisted surgery appears to offer the bridge between improved patient perioperative outcomes and surgeon ergonomics. With our current knowledge and experience, it appears that laparoscopy and robotic-assisted surgery are valuable in the management of cervical, uterine, and ovarian cancers, whereas all methods of minimally invasive surgery (laparoscopy, robotic-assistance, and LESS) are useful for prophylactic bilateral salpingo-oophorectomy and/or adnexal surgery. The long-term oncologic data remains to be determined with these procedures but is unlikely to differ greatly from the laparoscopic outcome data that we currently have. As we continue to develop new surgical techniques such as LESS, we may be able to even further decrease the risk of postoperative complications and improve patient satisfaction. At the core of these developments lies improved oncologic outcomes and improved patient satisfaction without compromising oncologic outcomes. As we continue to utilize minimally invasive surgery to care for the gynecologic oncology patient, we should also look at oncologic outcomes to prove that they are not inferior to the outcomes of patients undergoing laparotomy. With every new advancement and novel technology or technique, it will be prudent for the gynecologic oncology society as a whole to carefully evaluate the risks and benefits of these procedures to our patients and to never overlook the individual-based treatment they deserve.
